# High-Performance Cathode Material of FeF_3_·0.33H_2_O Modified with Carbon Nanotubes and Graphene for Lithium-Ion Batteries

**DOI:** 10.1186/s11671-019-2925-y

**Published:** 2019-03-15

**Authors:** Lu Lu, Sheng Li, Jun Li, Lifang Lan, Yan Lu, Shuaijun Xu, Si Huang, Chunyang Pan, Fenghua Zhao

**Affiliations:** School of Chemical Engineering and Light Industry, Guangdong University of Technology, No. 100 Waihuan xi Road, Guangzhou Higher Education Mega Center, Panyu District, Guangzhou, 510006 China

**Keywords:** FeF_3_·0.33H_2_O cathode material, Conductive network, Electrochemical performance, Lithium-ion batteries

## Abstract

The FeF_3_·0.33H_2_O cathode material can exhibit a high capacity and high energy density through transfer of multiple electrons in the conversion reaction and has attracted great attention from researchers. However, the low conductivity of FeF_3_·0.33H_2_O greatly restricts its application. Generally, carbon nanotubes (CNTs) and graphene can be used as conductive networks to improve the conductivities of active materials. In this work, the FeF_3_·0.33H_2_O cathode material was synthesized via a liquid-phase method, and the FeF_3_·0.33H_2_O/CNT + graphene nanocomposite was successfully fabricated by introduction of CNTs and graphene conductive networks. The electrochemical results illustrate that FeF_3_·0.33H_2_O/CNT + graphene nanocomposite delivers a high discharge capacity of 234.2 mAh g^−1^ in the voltage range of 1.8–4.5 V (vs. Li^+^/Li) at 0.1 C rate, exhibits a prominent cycling performance (193.1 mAh g^−1^ after 50 cycles at 0.2 C rate), and rate capability (140.4 mAh g^−1^ at 5 C rate). Therefore, the electronic conductivity and electrochemical performance of the FeF_3_·0.33H_2_O cathode material modified with CNTs and graphene composite conductive network can be effectively improved.

## Introduction

Rechargeable lithium-ion batteries (LIBs) are the most effective power storage systems for portable electronic devices and considered as promising candidates for electric vehicles (EVs) and hybrid electric vehicles (HEVs) [1]. Compared with traditional fossil energy, LIBs are renewable and clean energy and friendly to the environment. Recently, with the rapid development of LIBs technology, demands for both energy and power density have continuously increased. One key challenge is developing high-performance electrode active materials, and the cathode material is a vital factor for improving the electrochemical properties of LIBs, including the specific capacity, cycling capability, rate capability, etc. [2, 3]. Commercialized cathode materials, such as LiCoO_2_ [4], LiMn_2_O_4_ [5], and LiFePO_4_ [6], suffer from low theoretical capacities due to the intercalation reaction involving only a single electron reaction, which cannot satisfy the demands of EVs. In the past several years, multi-electron materials have attracted substantial interest because they can realize the transfer of more than one electron through the conversion reaction [7]. Metal fluorides are ideal cathode materials with high theoretical capacities, energy densities, and operating voltages. Among them, FeF_3_ has been regarded as the most suitable cathode material due to its high theoretical specific capacity of 712 mAh g^−1^ (3e^−^ transfer) and 237 mAh g^−1^ (1e^−^ transfer), high discharge voltage plateau at approximately 2.7 V, and superb thermal stability [8–10].

Despite these remarkable merits, FeF_3_ as a cathode material still has several shortcomings, which have restricted its practical application. The main drawback of FeF_3_ is its electronic insulating behavior caused by a high ionicity, which induces a large band gap of the Fe-F bond and eventually leads to a low actual specific capacity, an inferior rate capability, and poor energy efficiency [11–13]. In order to resolve these issues, various strategies have been adopted to overcome the poor electronic and ionic conductivities. Generally, the methods to improve conductivity can be summarized in three aspects as follows: (1) element doping. Element doping can effectively decrease the band gap and actively effect microcrystal growth [14, 15]. Rahman et al. prepared Co-doped iron fluoride (Fe_0.9_Co_0.1_F_3_·0.5H_2_O) by a non-aqueous precipitation method, resulting in a high discharge capacity of 227 mAh g^−1^ at 0.1 C between 1.8 and 4.5 V [14]. (2) Surface coating. Modification by introducing a coating layer can significantly shorten the Li^+^ transport length and alleviate volume changes [16]. Ma et al. successfully fabricated FeF_3_ coated with poly (3,4-ethylenedioxythiophene) (PEDOT) via a novel in situ polymerization method, and the sample exhibited a high power capability of 120 mAh g^−1^ at 1 A g^−1^ at room temperature due to the improved ionic and electronic transport in the electrode [17]. (3) Fabricating composite with conductive additives. It can substantially enhance the cycling and rate performance of the FeF_3_ cathode material [18–21]. Jung et al. obtained FeF_3_/ordered mesoporous carbon (OMC) nanocomposite that showed a high reversible specific capacity (178 mAh g^−1^ at 0.1 C during the second cycle in the voltage range of 2.0–4.5 V) and better cycling stabilities (capacity fading of 8.8%) than bulk FeF_3_ (capacity fading of approximately 42%) at 30 cycles [22]. Noticeably, the fabrication of composite with conductive network is the most beneficial approach to improve both the ionic and electronic conductivities to eventually enhance the electrochemical performance of the cathode material.

Iron(III) fluoride cathode materials with different amounts of hydration water, for example, FeF_3_·0.33H_2_O [23], FeF_3_·0.5H_2_O [24], FeF_3_·3H_2_O [25], and FeF_3_ [26], have been extensively reported. Among them, hexagonal tungsten bronze-type FeF_3_·0.33H_2_O demonstrated that with the best electrochemical property, its characteristic one-dimensional hexagonal cavity is convenient for efficient Li^+^ transport and can facilitate electrolyte penetration [27]. In addition, the unique structure can effectively limit the movement of water and stabilize the crystal structure. Different functionalized carbon matrixes have been used as conductive networks, but overall, carbon nanotubes (CNTs) and graphene exhibit significant potential as conductive medium due to their distinguished electronic conductivities and excellent stabilities [28–31]. Graphene, with its large specific area, can promote sufficient contact at the electrode and electrolyte interface, and the graphene network plays an important role in electron transfer and ion migration. Furthermore, graphene provides excellent mechanical stability, which contributes to the bend and stretch of electrode [32, 33].

In this study, nanostructured FeF_3_·0.33H_2_O cathode material was synthesized via a liquid-phase method, and then, the precursor was milled with CNTs followed by sintering to obtain FeF_3_·0.33H_2_O/CNT composite that was further mixed with graphene conducting paste without a binder. Finally, the CNTs and graphene co-modified FeF_3_·0.33H_2_O nanocomposite was successfully prepared. The CNTs with intrinsic flexibility and large specific surface area can greatly facilitate the electron transport, and graphene with high mechanical strength and high chemical stability can effectively buffer the volume change and provides a support for electrochemical reaction [31, 34, 35]. Moreover, the interconnecting of CNTs and graphene sheets can construct an integrated three-dimensional conductive framework, which tremendously promotes Li^+^ diffusion and simultaneously increases the structure stability. Therefore, compared to the FeF_3_·0.33H_2_O composite with a single conductive network of CNTs and pure FeF_3_·0.33H_2_O, FeF_3_·0.33H_2_O nanocomposite with CNTs and graphene networks exhibits superior electrochemical properties. The morphologies, crystal structures, and electrochemical performances of all the samples were systematically investigated.

## Results and Discussion

### Structural and Morphology Analysis

**Fig. 1:**
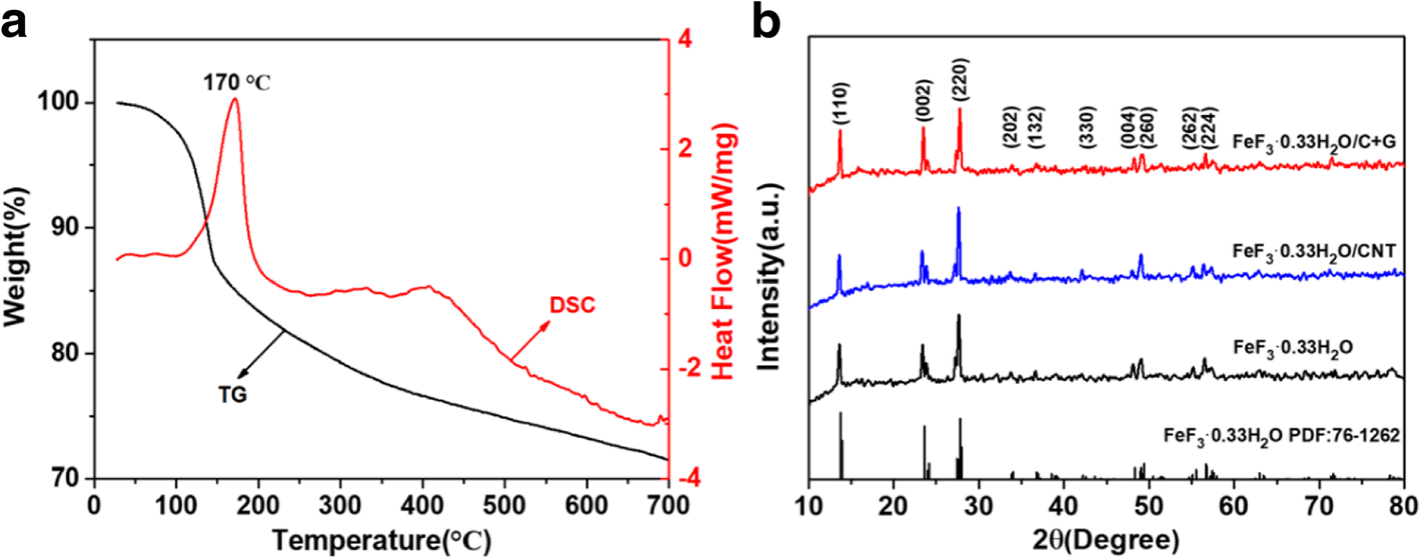
**a** TG-DSC curve of the FeF_3_·3H_2_O precursor measured from 30 to 700 °C at a heating rate of 10 °C min^−1^ under an argon atmosphere. **b** XRD patterns of FeF_3_·0.33H_2_O, FeF_3_·0.33H_2_O/CNT, and FeF_3_·0.33H_2_O/C + G

Thermogravimetric-differential scanning calorimetry (TG-DSC) measurement was carried out to confirm the dehydration temperature of the FeF_3_·3H_2_O precursor and the result is shown in Fig. 1a. Four stages of the weight loss curve are found in the regions of 30–110 °C, 110–250 °C, 250–450 °C, and 450–700 °C. In the first stage of 30–110 °C, a slight weight loss of approximately 3% can be attributed to phase transformation of crystal. In the second stage of 110–250 °C, the TG curve has a rapid weight loss of about 15% and the DSC curve shows an evident endothermic peak around 170 °C; the corresponding reaction process is the removal of hydration water (2.67 H_2_O) from FeF_3_·3H_2_O. In the third stage of 250–450 °C, the weight loss is about 6% which may be due to the removal of hydration water for FeF_3_·0.33H_2_O transforming to FeF_3_ and a weak exothermic peak is observed from the DSC curve. In the last stage of 450–700 °C, a little weight loss of about 4% is probably due to the decomposition of FeF_3_. According to these results, the precursor was dried at 80 °C in a vacuum oven to remove the absorbed water and calcinated at 240 °C to obtain FeF_3_·0.33H_2_O.

X-ray diffraction (XRD) measurements were conducted to investigate the crystal structure of the synthesized samples. The XRD patterns of FeF_3_·0.33H_2_O, FeF_3_·0.33H_2_O/CNT, and FeF_3_·0.33H_2_O/C + G are depicted in Fig. 1b. All samples reveal diffraction peaks positioned at 2θ = 13.79°, 23.62°, and 27.80° in accordance with the (110), (002), and (220) facets, which matched well with the standard spectrum of hexagonal tungsten bronze structure FeF_3_·0.33H_2_O (PDF No. 76-1262) [36]. No evident characteristic peak of CNTs and graphene are observed in the XRD pattern of the FeF_3_·0.33H_2_O/CNT and FeF_3_·0.33H_2_O/C + G samples, which is mainly due to the low contents of CNTs and graphene.

**Fig. 2:**
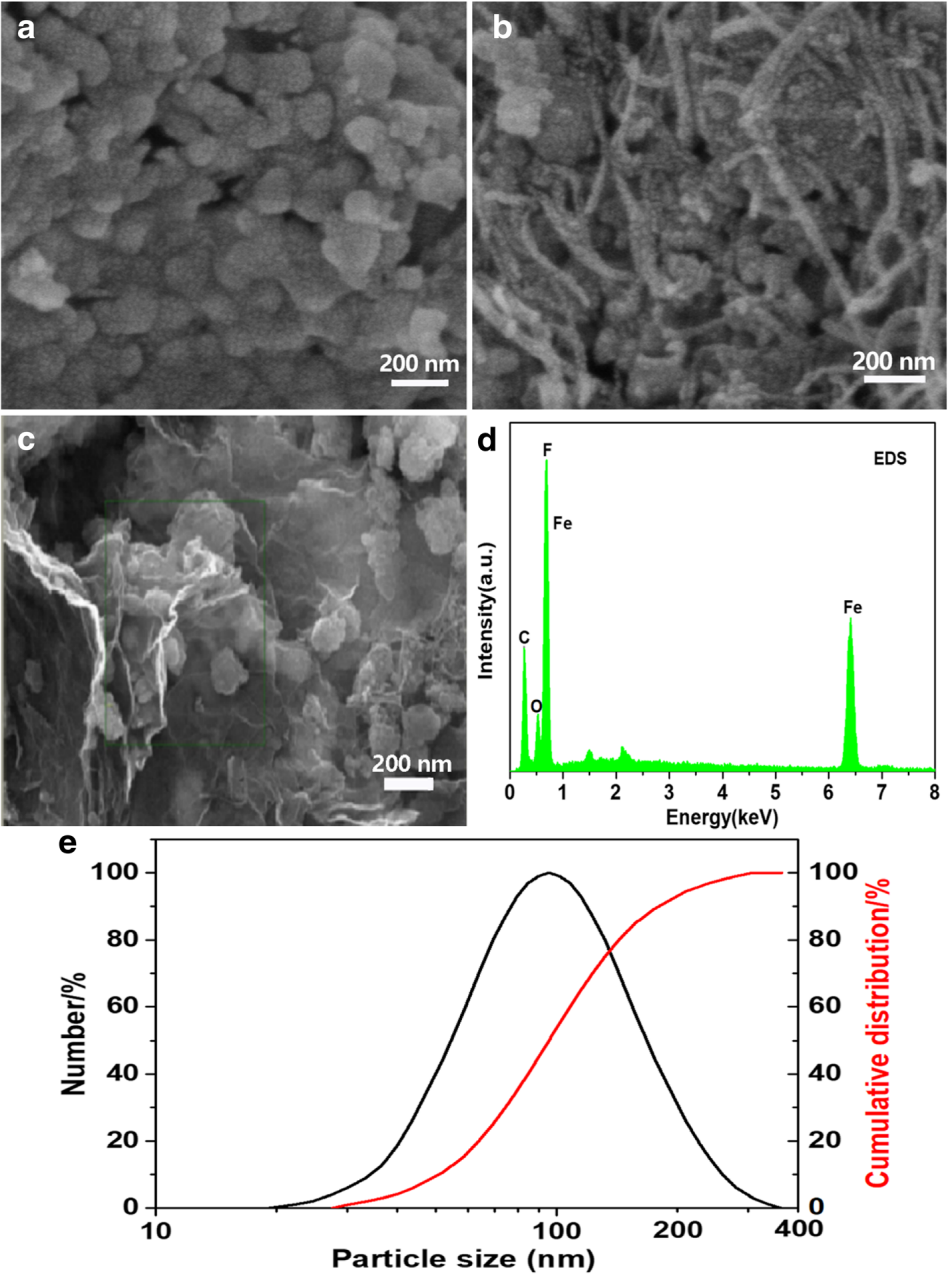
SEM images, **a** FeF_3_·0.33H_2_O, **b** FeF_3_·0.33H_2_O/CNT, and **c** FeF_3_·0.33H_2_O/C + G. **d** EDS of the FeF_3_·0.33H_2_O/C + G. **e** Particle size distribution diagram of FeF_3_·0.33H_2_O

The SEM and EDS measurements were performed to analyze the microstructure of the composites. The morphologies and particle sizes of FeF_3_·0.33H_2_O, FeF_3_·0.33H_2_O/CNT and FeF_3_·0.33H_2_O/C + G nanocomposites are shown in Fig. 2. As distinctly seen from Fig. 2a, the particle size of pure FeF_3_·0.33H_2_O is around 100 nm, and the particles are uniform in size and well distributed, slight aggregation is observed, and the particle size of FeF_3_·0.33H_2_O can be further confirmed by particle size distribution diagram shown in Fig. 2e. Figure 2b presents the morphology of FeF_3_·0.33H_2_O/CNT nanocomposite. Clearly, the conductive network of CNTs is intimately intertwined on the surface of the FeF_3_·0.33H_2_O particles. For the FeF_3_·0.33H_2_O/C + G nanocomposite, the surface of the FeF_3_·0.33H_2_O particles is wrapped by CNTs and graphene sheets; as shown in Fig. 2c, the FeF_3_·0.33H_2_O particles and CNTs are well covered by graphene sheets. In addition, the graphene sheets are preserved well-layered structure in the FeF_3_·0.33H_2_O/C + G nanocomposite, which can provide a fast channel for Li^+^ transport. The conductive contact between the FeF_3_·0.33H_2_O material and current collector can be significantly improved by CNTs and graphene due to their outstanding electronic conductivity. Especially graphene with a large surface area can provide an additional transport channel for Li-ion diffusion, which makes the FeF_3_·0.33H_2_O/C + G nanocomposite with superior electrochemical performance. EDS test was carried out to further investigate the elemental composition of the FeF_3_·0.33H_2_O/C + G nanocomposite. The elements of Fe, F, O, and C can be observed from the EDS image in Fig. 2d.

**Fig. 3:**
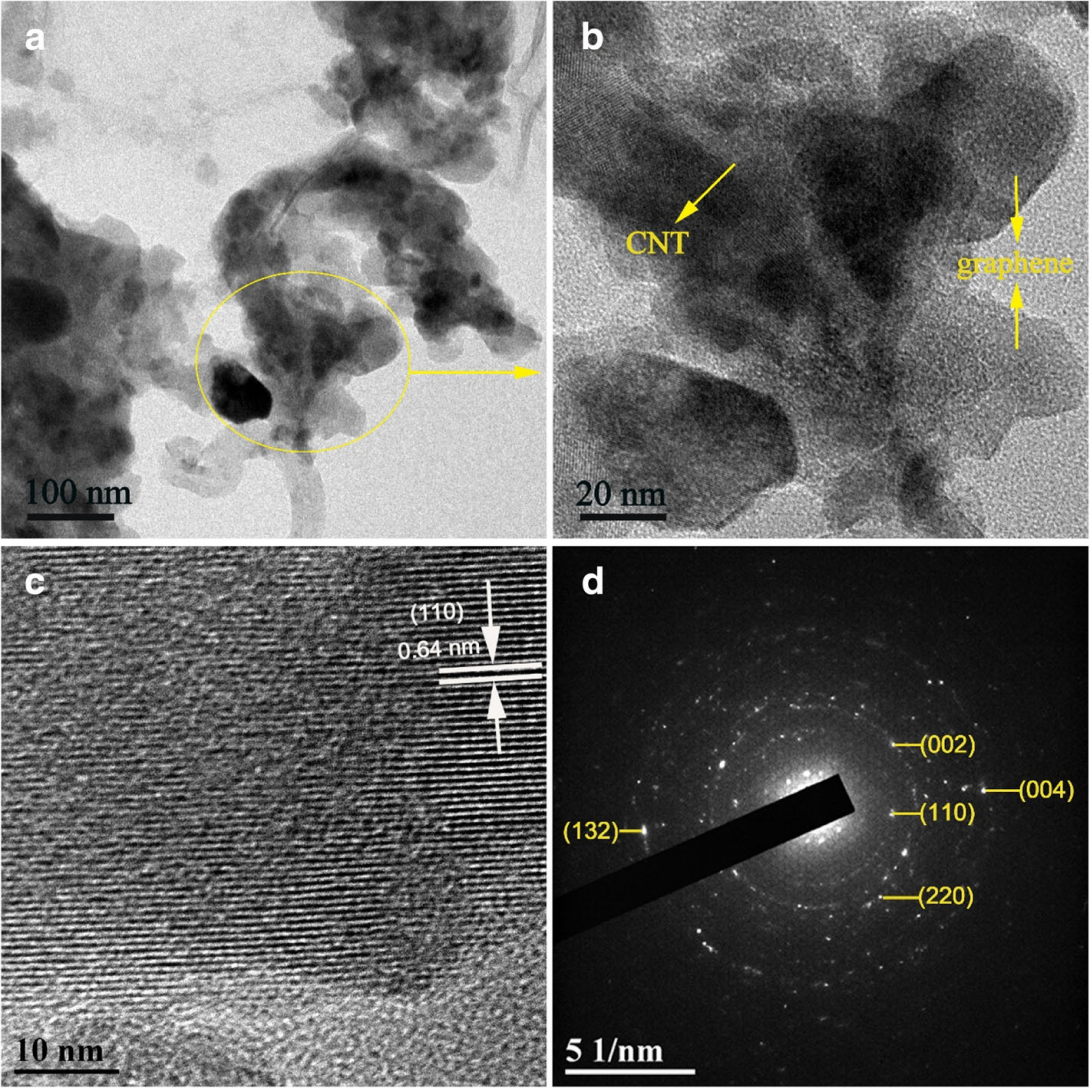
**a**, **b** TEM images of FeF_3_·0.33H_2_O/C + G nanocomposite. **c** HRTEM image of FeF_3_·0.33H_2_O/C + G nanocomposite. **d** SAED image of FeF_3_·0.33H_2_O/C + G nanocomposite

The morphology and detailed microstructure of the FeF_3_·0.33H_2_O/C + G nanocomposite were further studied by TEM, and the TEM images are displayed in Fig. 3. As shown in Fig. 3a, b, the FeF_3_·0.33H_2_O particles and CNTs and graphene sheets are closely interconnected with each other, which is consistent with the result of the SEM images. The HRTEM image shown in Fig. 3c offers no evident delineation between the bulk and wrapping layer; the lattice fringe spacing of 0.64 nm coincides with the (110) facet of FeF_3_·0.33H_2_O. The SAED pattern of the FeF_3_·0.33H_2_O/C + G nanoparticle is shown in Fig. 3d; the planes of (110), (002), (220), (132), and (004) correspond to the XRD results, which indexed to the hexagonal tungsten bronze structure FeF_3_·0.33H_2_O. The FeF_3_·0.33H_2_O/C + G nanocomposite with small particle size and superb conductive network structure, favors sufficient contact between the electrode material and electrolyte and facilitates Li-ion transport; therefore, better electrochemical performance can be achieved.

### Electrochemical Characterization

**Fig. 4:**
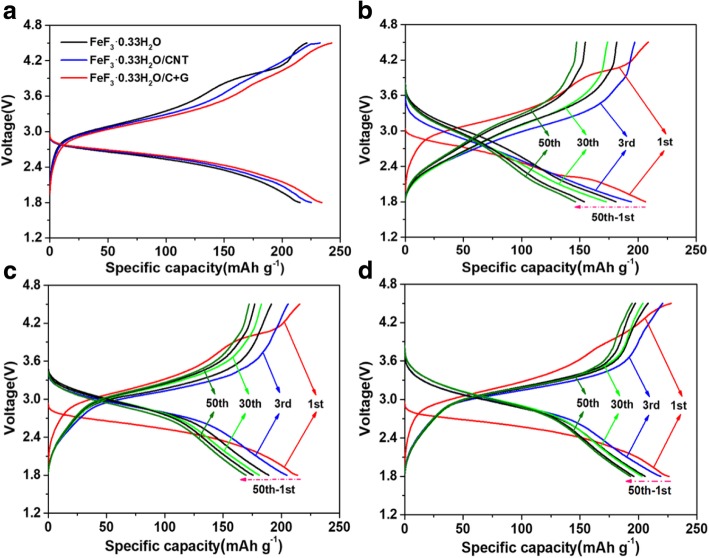
**a** The initial charge-discharge profiles of FeF_3_·0.33H_2_O, FeF_3_·0.33H_2_O/CNT, and FeF_3_·0.33H_2_O/C + G electrodes at 0.1 C rate. Charge and discharge profiles of different cycles (1st, 3rd, 30th, 50th) at 0.2 C rate, **b** FeF_3_·0.33H_2_O, **c** FeF_3_·0.33H_2_O/CNT, and **d** FeF_3_·0.33H_2_O/C + G

To investigate the electrochemical properties of all samples, galvanostatic charge/discharge tests were implemented in the voltage range of 1.8–4.5 V (vs. Li^+^/Li), and this voltage range allows only one electron reaction to occur. The charge-discharge profiles of all samples are shown in Fig. 4. The initial charge-discharge curves of the three electrodes at 0.1 C (1 C = 237 mAh g^−1^) rate are shown in Fig. 4a; the pristine FeF_3_·0.33H_2_O electrode exhibits the lowest initial discharge capacity of 217.5 mAh g^−1^, which may be due to the poor electronic conductivity of FeF_3_·0.33H_2_O. While the FeF_3_·0.33H_2_O/CNT and FeF_3_·0.33H_2_O/C + G electrodes deliver higher initial discharge capacities of approximately 225.1 mAh g^−1^ and 234.2 mAh g^−1^, respectively. In our test, the initial discharge capacity of the FeF_3_·0.33H_2_O/C + G electrode is only 16.7 mAh g^−1^ higher than that of the pristine FeF_3_·0.33H_2_O electrode, illustrating CNTs and graphene almost deliver no capacity in the FeF_3_·0.33H_2_O/C + G sample. The slightly increased capacity can be attributed to the CNTs and graphene incorporation enhanced the electron transport and reduced electrochemical polarization. From the initial charge-discharge curves of all electrodes, all the curves have an evident discharge plateau at 2.7 V due to the insertion reaction (Li^+^ + *e*^−^ + FeF_3_ ∙ 0.33H_2_O → LiFeF_3_ ∙ 0.33H_2_O). The charge-discharge profiles of different cycles at 0.2 C rate are presented in Fig. 4b–d. As shown in Fig. 4b, the FeF_3_·0.33H_2_O electrode only delivers a capacity of 146.2 mAh g^−1^ at 0.2 C rate after 50 cycles. The FeF_3_·0.33H_2_O/CNT electrode delivers a capacity of 170.3 mAh g^−1^ after 50 cycles shown in Fig. 4c. It is worth noting that the FeF_3_·0.33H_2_O/C + G electrode still retain a capacity of 193.1 mAh g^−1^ even after 50 cycles shown in Fig. 4d. In addition, the FeF_3_·0.33H_2_O/C + G electrode presents the lowest charge voltage plateau and the highest discharge voltage plateau, demonstrating that it has the smallest electrochemical polarization and excellent reversibility, thus mitigate the voltage hysteresis. The better performance of FeF_3_·0.33H_2_O/CNT and FeF_3_·0.33H_2_O/C + G electrodes demonstrate that adding CNTs and graphene can effectively improve the conductivity of the FeF_3_·0.33H_2_O cathode material. Particularly, the FeF_3_·0.33H_2_O/C + G electrode exhibits the best electrochemical performance due to the interlacing of CNTs and graphene forms a three-dimensional conductive structure, which tremendously facilitate the transport of Li-ion, and thus resulting in promoting the intercalation process of Li-ions [37, 38].

**Fig. 5:**
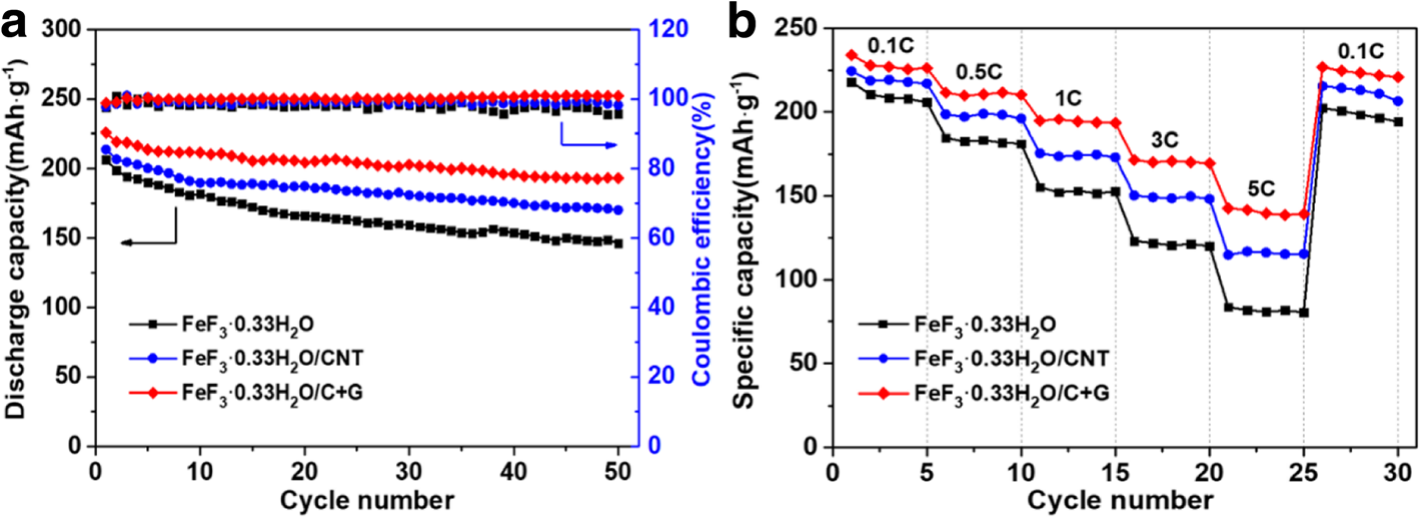
**a** Cycling performances of FeF_3_·0.33H_2_O, FeF_3_·0.33H_2_O/CNT, and FeF_3_·0.33H_2_O/C + G electrodes at 0.2 C rate. **b** Rate performances of FeF_3_·0.33H_2_O, FeF_3_·0.33H_2_O/CNT, and FeF_3_·0.33H_2_O/C + G electrodes at different current density

To further demonstrate the excellent cycling stability of the FeF_3_·0.33H_2_O/C + G nanocomposite, the cycling capabilities of the FeF_3_·0.33H_2_O, FeF_3_·0.33H_2_O/CNT, and FeF_3_·0.33H_2_O/C + G electrodes up to the 50th cycle at 0.2 C rate in the voltage range of 1.8–4.5 V (vs. Li^+^/Li) are shown in Fig. 5a. The FeF_3_·0.33H_2_O electrode with a rapid capacity decay and a poor capacity retention of approximately 70.83% (capacity fading rate of 0.58% per cycle) after 50 cycles. The FeF_3_·0.33H_2_O/CNT electrode shows a capacity retention of about 79.65% (0.41% fading per cycle) after 50 cycles. Notably, the FeF_3_·0.33H_2_O/C + G electrode achieve the highest capacity retention of 85.48% (only 0.29% fading per cycle) after 50 cycles. Moreover, the coulombic efficiency of the FeF_3_·0.33H_2_O/C + G electrode can reach up to over 99% during the Li^+^ insertion and extraction processes. The above results demonstrate that CNTs and graphene can improve the electronic conductivity and enhance the discharge capacities of FeF_3_·0.33H_2_O. Particularly, the FeF_3_·0.33H_2_O/C + G electrode exhibits the best cycling performance, illustrating higher electrical conductivity, better reversibility, and lower polarization after adding of CNTs and graphene. CNTs with high surface area supply sufficient pathway for electron transfer and the graphene works as an excellent conductive network for enabling fast Li^+^ transport between the electrolyte and electrode [28, 35]. Moreover, the FeF_3_·0.33H_2_O particles and CNTs can work as spacers to impede the stacking of graphene sheets and thus provide high active surface area. Therefore, the interaction of FeF_3_·0.33H_2_O particles and CNTs and graphene can significantly improve the cycling performance.

The rate capabilities of the FeF_3_·0.33H_2_O, FeF_3_·0.33H_2_O/CNT, and FeF_3_·0.33H_2_O/C + G electrodes were evaluated at 0.1 C, 0.5 C,1 C, 3 C, and 5 C rates and then again at 0.1 C rate and results are displayed in Fig. 5b. The discharge capacities of all samples are decreased with increased current density. As expected, the FeF_3_·0.33H_2_O/C + G electrode presents a superior rate performance among the three electrodes and delivers average discharge capacities of 228 mAh g^−1^, 210.7 mAh g^−1^, 194.4 mAh g^−1^, 170.5 mAh g^−1^, and 140.4 mAh g^−1^ at 0.1 C, 0.5 C, 1 C, 3 C, and 5 C rates. When the rate is returned to 0.1 C, the electrode can still deliver a discharge capacity of 226.7 mAh g^−1^. For comparison, FeF_3_·0.33H_2_O and FeF_3_·0.33H_2_O/CNT electrodes show inferior rate performance; they deliver poor discharge capacities of 81.7 mAh g^−1^ and 115.7 mAh g^−1^ at 5 C rate, which are remarkably lower than that of FeF_3_·0.33H_2_O/C + G electrode. As a result, the rate capability of the FeF_3_·0.33H_2_O/C + G electrode is significantly improved compared to those of FeF_3_·0.33H_2_O without or with a single CNT conductive network. Therefore, the good rate capability of the FeF_3_·0.33H_2_O/C + G electrode result from the CNTs and graphene conductive networks, which enhanced the electronic conductivity, and above all, the constructed three-dimensional conductive network is beneficial for Li-ion insertion and extraction between electrodes.

**Fig. 6:**
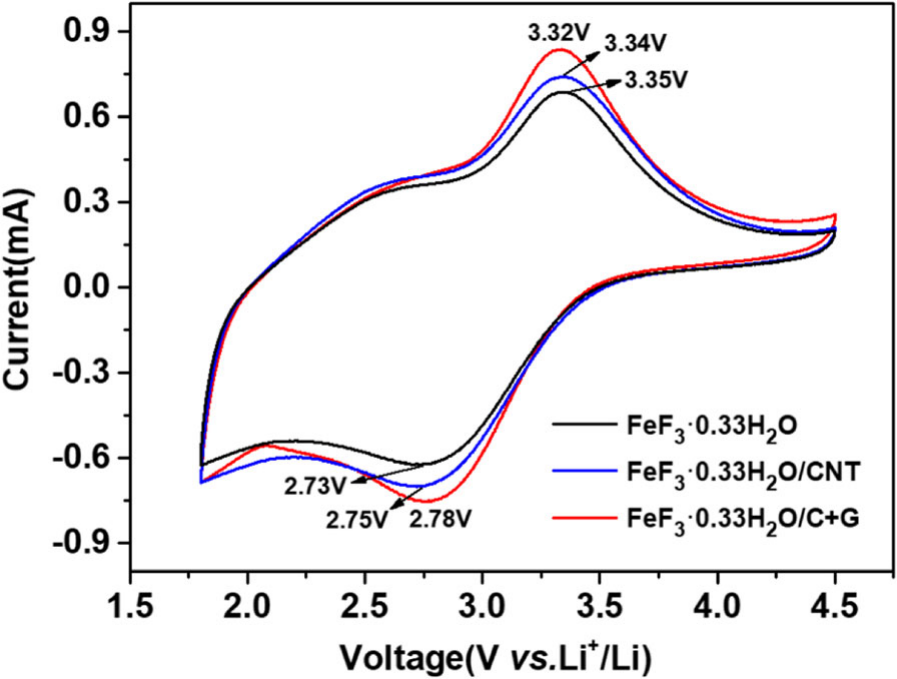
CV curves of FeF_3_·0.33H_2_O, FeF_3_·0.33H_2_O/CNT, and FeF_3_·0.33H_2_O/C + G electrodes at a scan rate of 1 mV s^−1^

Cyclic voltammogram (CV) measurements were carried out to further examine the electrochemical properties of the FeF_3_·0.33H_2_O, FeF_3_·0.33H_2_O/CNT, and FeF_3_·0.33H_2_O/C + G electrodes at a scan rate of 1 mV s^−1^ between 1.8 V and 4.5 V (vs. Li^+^/Li) which are shown in Fig. 6. The three curves display similar shapes with apparent oxidation/reduction peaks corresponding to delithiation/lithiation processes. The oxidation and reduction peaks of the FeF_3_·0.33H_2_O/C + G electrode are detected at 3.32 V and 2.78 V, and the potential interval (*ΔE*_p_) is 0.54 V. While the *ΔE*_p_ values of the FeF_3_·0.33H_2_O and FeF_3_·0.33H_2_O/CNT electrodes are 0.59 V and 0.62 V, respectively. Smaller *ΔE*_p_ value indicates a smaller electrochemical polarization and a better reversibility of the electrode. In addition, the FeF_3_·0.33H_2_O/C + G electrode exhibits a higher current and a larger area than those of the FeF_3_·0.33H_2_O and FeF_3_·0.33H_2_O/CNT electrodes. The area surrounded by the CV curve represents the capacity of the material; the larger area is related to the higher capacity, and the change rate of area represents the decay rate of capacity. The results reveal that the FeF_3_·0.33H_2_O/C + G electrode has a higher capacity and better reversibility, which is well consistent with the galvanostatic charge/discharge tests.

**Fig. 7:**
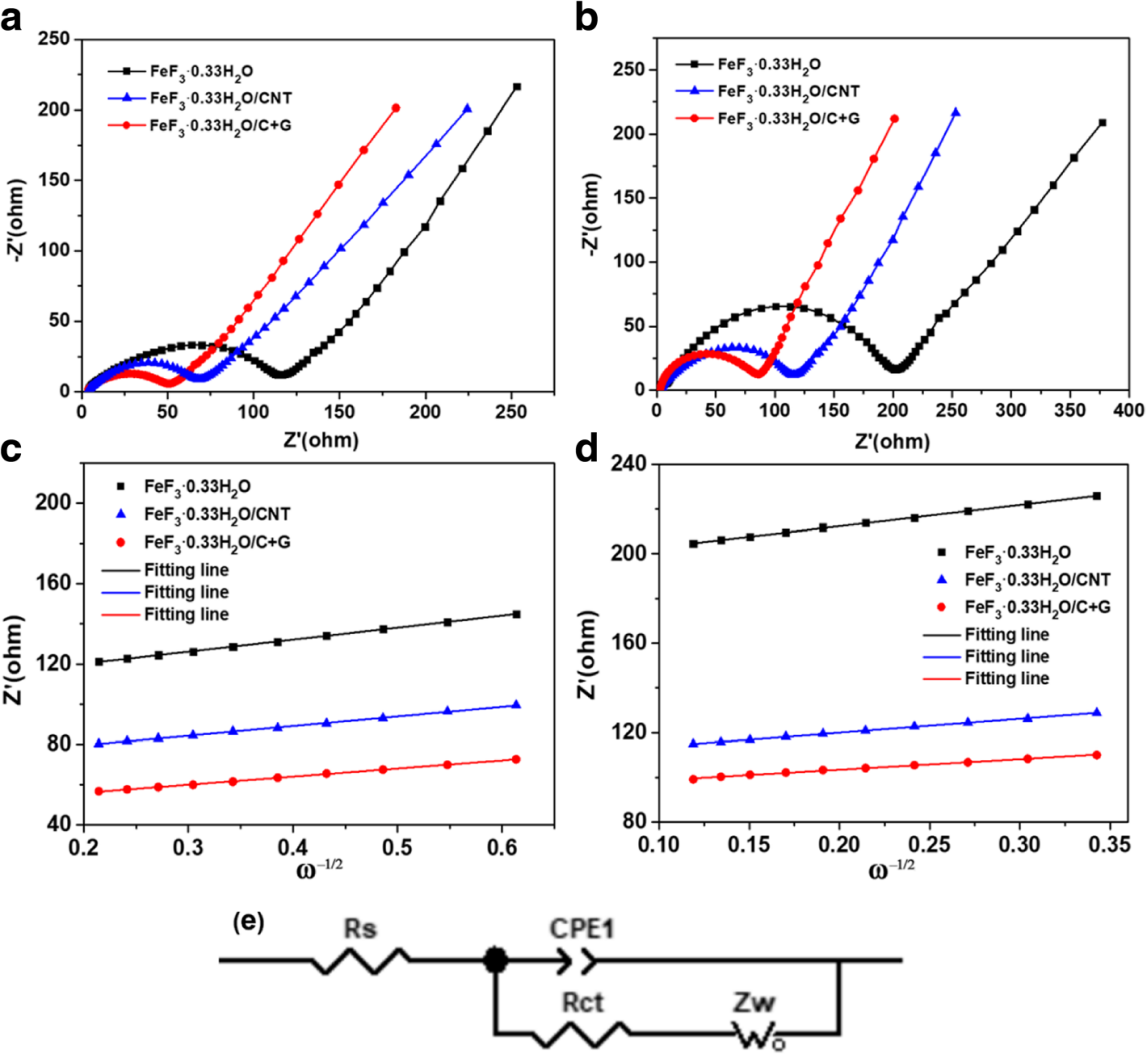
The Nyquist plots of FeF_3_·0.33H_2_O, FeF_3_·0.33H_2_O/CNT, and FeF_3_·0.33H_2_O/C + G electrodes; **a** the 3rd cycle and **b** the 50th cycle. The relationship plots of FeF_3_·0.33H_2_O, FeF_3_·0.33H_2_O/CNT, and FeF_3_·0.33H_2_O/C + G electrodes between *Z’* and *ω*^−1/2^ at low-frequency region; **c** the 3rd cycle and **d** the 50th cycle. **e** The corresponding equivalent circuit model of EIS

Electrochemical impedance spectroscopy (EIS) measurements were performed to explore the electrochemical reaction kinetics behavior of the FeF_3_·0.33H_2_O, FeF_3_·0.33H_2_O/CNT, and FeF_3_·0.33H_2_O/C + G electrodes after the 3rd cycle and 50th cycle, and results are shown in Fig. 7a, b. All the Nyquist plots of the electrodes after activation are consisted of a semicircle and a sloping line. The semicircle in the high frequency is related to the charge transfer resistance (*R*_ct_), which represents the reaction kinetics of the electrode. The smaller radius of semicircle demonstrates the easier transport of Li^+^ and the electron transfer between the electrolyte and electrode interface, and the sloping line in the low frequency is associated with the Warburg resistance (*Z*_w_) of Li^+^ diffusion in the bulk of cathode material [39]. The corresponding equivalent circuit model was constructed to illustrate the impedance spectra shown in Fig. 7e; the uncompensated ohmic resistance (*R*_s_) represents the resistance of the electrolyte and electrode material, and the constant phase-angle element (CPE) represents the double-layer capacitance and passive film capacitance [40]. The impedance values of *R*_s_ and *R*_ct_ for the three electrodes after the 3rd and 50th cycle are listed in Table 1. No significant difference of *R*_s_ values for the three electrodes after the 3rd cycle is noted. However, the *R*_ct_ value (50.9 Ω) of the FeF_3_·0.33H_2_O/C + G electrode is evidently lower than those of the FeF_3_·0.33H_2_O (115.7 Ω) and FeF_3_·0.33H_2_O/CNT (68.2 Ω) electrodes, which indicated less polarization of the FeF_3_·0.33H_2_O/C + G electrode. Moreover, the *R*_ct_ value of the FeF_3_·0.33H_2_O/C + G electrode is 86.5 Ω after the 50th cycle, which is also the smallest among the three electrodes. The lower *R*_ct_ value of the electrode after activation suggested better charge transfer kinetics behavior. The lithium ion diffusion coefficients (D_Li+_) of the FeF_3_·0.33H_2_O, FeF_3_·0.33H_2_O/CNT, and FeF_3_·0.33H_2_O/C + G electrodes are calculated from the following equation [41],

1 $$ {D}_{\mathrm{Li}+}=\frac{{\mathrm{R}}^2{\mathrm{T}}^2}{2{\mathrm{A}}^2{\mathrm{n}}^4{\mathrm{F}}^4{\mathrm{C}}^2{\upsigma}_{\upomega}^2} $$

**Table 1:** *R*_s_, *R*_ct_, and *D*_Li+_ values of FeF_3_·0.33H_2_O, FeF_3_·0.33H_2_O/CNT, and FeF_3_·0.33H_2_O/C + G electrodes after the 3rd and 50th cycle

Samples	3rd cycle			50th cycle		
	*R*_s_ (Ω)	*R*_ct_ (Ω)	*D*_Li+_ (cm^2^ s^−1^)	*R*_s_ (Ω)	*R*_ct_ (Ω)	*D*_Li+_ (cm^2^ s^−1^)
FeF_3_·0.33H_2_O	4.2	115.7	7.63 × 10^−13^	8.2	202.6	2.96 × 10^−13^
FeF_3_·0.33H_2_O/CNT	4.0	68.2	1.19 × 10^−12^	4.2	115.7	7.10 × 10^−13^
FeF_3_·0.33H_2_O/C + G	3.8	50.9	1.67 × 10^−12^	3.9	86.5	1.21 × 10^−12^

Equation 1 calculate the diffusion coefficient of Li^+^

Equation 2 a relationship between *σ*_ω_ and *Z’*

In Eq. (1), *R* is the gas constant, *T* is the absolute temperature, *A* is the surface area of electrode, *n* is the number of electrons involved in the redox reaction, *F* is the Faraday constant, *C* is the molar concentration of Li^+^, and *σ*_ω_ is the Warburg coefficient which can be obtained from the following relationship,

2 $$ {\mathrm{Z}}^{\hbox{'}}={R}_{\mathrm{s}}+{R}_{\mathrm{ct}}+{\upsigma}_{\upomega}{\upomega}^{-1/2} $$

where *Z’* is the real part of impedance and *ω* is the angular frequency in the low-frequency region. The linearity of *Z’* and *ω*^− 1/2^ after the 3rd cycle and 50th cycle are shown in Fig. 7c, d. The Li^+^ diffusion coefficients of the three electrodes are listed in Table 1. The *D*_Li+_ value (1.67 × 10^−12^ cm^2^ s^−1^) of the FeF_3_·0.33H_2_O/C + G electrode after the 3rd cycle is higher than those of the FeF_3_·0.33H_2_O/CNT (1.19 × 10^−12^ cm^2^ s^−1^) and FeF_3_·0.33H_2_O (7.63 × 10^−13^ cm^2^ s^−1^). In addition, the *D*_Li+_ values of the FeF_3_·0.33H_2_O, FeF_3_·0.33H_2_O/CNT, and FeF_3_·0.33H_2_O/C + G electrodes after the 50th cycle are 2.96 × 10^−13^ cm^2^ s^−1^, 7.10 × 10^−13^ cm^2^ s^−1^, and 1.21 × 10^−12^ cm^2^ s^−1^, respectively. Apparently, the *D*_Li+_ values of the FeF_3_·0.33H_2_O/C + G electrode are the highest among the three electrodes, indicating that the FeF_3_·0.33H_2_O/C + G shows better electrode reaction kinetics. The results confirm that the conductive network constructed by CNTs and graphene can effectively reduce the polarization of FeF_3_·0.33H_2_O/C + G electrode, which contribute to excellent electrochemical performance.

## Conclusions

In summary, the FeF_3_·0.33H_2_O cathode material was successfully synthesized by a liquid-phase method, and the FeF_3_·3H_2_O precursor was milled with CNTs conductive network followed by sintering to obtain FeF_3_·0.33H_2_O/CNT nanocomposite, and then mixed with graphene conducting paste without a binder to obtain the FeF_3_·0.33H_2_O/C + G nanocomposite. The functional network consisted of CNTs and graphene provides an effective strategy to improve the electronic conductivity of FeF_3_·0.33H_2_O cathode material. The FeF_3_·0.33H_2_O/C + G nanocomposite exhibits better electrochemical performances with increased specific capacity, extended cyclic lifespan, and enhanced rate capability than that of pure FeF_3_·0.33H_2_O. The EIS results also indicate that the FeF_3_·0.33H_2_O/C + G electrode has the best electrochemical reaction kinetics behavior. The outstanding electrochemical performances of FeF_3_·0.33H_2_O/C + G can be attributed to the constructed three-dimensional conductive networks by CNTs and graphene, improving the electronic conductivity, facilitating the Li^+^ and electron transport, thus enhancing the cycling and rate capabilities. Therefore, the FeF_3_·0.33H_2_O cathode material modified with CNTs and graphene showed excellent electrochemical properties and exhibited great promise as a cathode material for LIBs application.

## Methods

### Synthesis of FeF_3_·0.33H_2_O Powder

FeF_3_·0.33H_2_O powder was synthesized via a liquid-phase method followed by an annealing treatment. For the synthesis of FeF_3_·0.33H_2_O powder, Fe(NO_3_)_3_·9H_2_O (Aladdin, 99.99%) and NH_4_F (Aladdin, 98%) were utilized as the iron and fluorine sources, respectively, and polyethylene glycol (PEG400, Aldrich, 20%) was used as a dispersant. First, 3.1 g Fe(NO_3_)_3_·9H_2_O was dissolved in 20 mL ethanol in a Teflon-lined stainless-steel autoclave, and then, three drops of PEG400 were added. Next, the solution was ultrasonicated for 10 min to obtain solution A. Then, 0.85 g NH_4_F was dissolved in 5 mL of deionized water and ultrasonicated to form solution B. Solution B was added dropwise into the constantly stirred solution A, and the yellow solution gradually became colorless, eventually gained conglobate precipitates. After stewing for 12 h at room temperature, the precipitates were alternately washed with deionized water and ethanol several times and then dried at 80 °C for 12 h in a vacuum oven. After cooling to room temperature naturally, the precipitates were ground into powder to obtain FeF_3_·3H_2_O precursor and then transferred into a tube furnace for calcination at 240 °C for 3 h under an argon atmosphere to remove the crystal water. Finally, the FeF_3_·0.33H_2_O powder was obtained.

### Preparation of FeF_3_·0.33H_2_O/CNT + Graphene Combination Electrode

To prepare FeF_3_·0.33H_2_O with CNTs and graphene conductive networks, optimized amount of 5 wt% CNTs were added into the as-prepared precursor, uniformly ground and heated at tube furnace (240 °C for 3 h) under an argon atmosphere to obtain FeF_3_·0.33H_2_O/CNT powder. Then, 0.5 g FeF_3_·0.33H_2_O/CNT powder was added into 1.5 mL graphene *N*-methyl pyrrolidinone paste (Aladdin, graphene content: 1–1 .5wt%), stirred 4 h to form a homogeneous slurry. The slurry was pasted on an Al foil and dried at 85 °C overnight to obtain the FeF_3_·0.33H_2_O/CNT + graphene (denoted as FeF_3_·0.33H_2_O/C + G) combination electrode. Notably, the process of making combination electrode did not require the addition of a binder.

### Characterization

Thermogravimetric-differential scanning calorimetry (TG-DSC) measurement of the precursor was carried out in the temperature range from 30 to 700 °C at a heating rate of 10 °C min^−1^ under an argon atmosphere. The crystal structures of all the samples were characterized by X-ray diffraction (XRD, Bruker AXS D8, Germany) with Cu Kα radiation in the 2θ range of 10°–80° at a scan rate of 8° min^−1^. The morphologies and particle sizes of the materials were observed by scanning electron microscopy (SEM, JEOL JSM-6610 LV) and energy-dispersive spectroscopy (EDS, JEOL JSM-6610 LV). Transmission electron microscopy (TEM) and selected area electron diffraction (SAED) were carried out to further investigate the microstructure of materials by using a transmission electron microscope (JEOL JSM-2100F).

### Electrochemical Measurement

The electrochemical performances of the prepared cathode materials were characterized by CR2032 coin-type half-cells. The working electrodes were made by mixing the cathode materials (FeF_3_·0.33H_2_O or FeF_3_·0.33H_2_O/CNT), carbon black (Super P Li carbon), and polyvinylidene fluoride (PVDF) at a weight ratio of 90:5:5 in *N*-methyl pyrrolidinone (NMP). When the slurry was stirred uniform, it was pasted on an Al foil and dried at 85 °C overnight. The FeF_3_·0.33H_2_O/C + G combination electrode was fabricated as mentioned above. The cathode electrodes were pressed and cut into several disks and weighted, and then they were dried at 85 °C for 4 h in a vacuum oven. The coin-type cells were assembled in an argon-filled glove box, where the oxygen and water contents were controlled to less than 0.1 ppm, metal Li foils as anodes and Celgard 2400 membrane as separator; 1.0 M LiPF_6_ in ethylene carbonate (EC), propylene carbonate (PC), and diethyl carbonate (DEC) with a volume ratio of 1:1:1 were used as electrolyte. All the coin cells were aged for 4 h before testing. Galvanostatic charge/discharge tests were performed in the voltage range of 1.8–4.5 V (vs. Li^+^/Li) on a Land battery test system (LAND CT-2001A, Wuhan, China) at room temperature. The specific capacities of the working electrodes were calculated based on the mass of the active cathode materials. Cyclic voltammetry (CV) and electrochemical impedance spectroscopy (EIS) were measured by an electrochemical workstation (CorrTest CS310). The scanning rate of the CV tests was 1 mV s^−1^ in the voltage range of 1.8–4.5 V (vs. Li^+^/Li). The frequency range of EIS was from 100 kHz to 0.01 Hz at potentiostatic signal amplitudes of 5 mV.

## Abbreviations

CNTs: Carbon nanotubes
CPE: Constant phase-angle element
CV: Cyclic voltammetry
DEC: Diethyl carbonate
D_Li+_: Lithium ion diffusion coefficients
EC: Ethylene carbonate
EDS: Energy-dispersive spectroscopy
EIS: Electrochemical impedance spectroscopy
EVs: Electric vehicles
FeF_3_·0.33H_2_O/C + G: FeF_3_·0.33H_2_O/CNT + graphene
HEVs: Hybrid electric vehicles
LIBs: Lithium-ion batteries
NMP: *N*-methyl pyrrolidinone
OMC: Ordered mesoporous carbon
PC: Propylene carbonate
PEDOT: poly (3,4-ethylenedioxythiophene)
PEG: Polyethylene glycol
PVDF: Polyvinylidene fluoride
*R*_ct_: Charge transfer resistance
*R*_s_: Ohmic resistance
SAED: Selected area electron diffraction
SEM: Scanning electron microscopy
TEM: Transmission electron microscopy
TG-DSC: Thermogravimetric-differential scanning calorimetry
XRD: X-ray diffraction
Z_w_: Warburg resistance
